# Amalgams—Their Uses and Abuses

**Published:** 1895-06

**Authors:** A. C. Hewitt

**Affiliations:** Chicago, Ill.


ARTICLE VI.

### AMALGAMS—THEIR USES AND ABUSES.

A. C. HEWITT, M. D., CHICAGO, ILL.

It needs no argument to prove to those who may chance to read this article, that dental amalgams have their uses. Nor is it necessary to argue that dental amalgams are abused. There is not a dentist by natural ability and special education equipped to practice his profession, with experience gained by an inspection of a hundred mouths, who will question either proposition. That the abuse is wide spread, and the victims to be numbered by the thousands and among the rich and educated, quite as often as with the poor and ignorant, is equally the shame and dishonor of the dental profession.

There seems to be a "Boston Rubber Conscience" governing the dialectics of many dentists, when they reason on the subject of amalgams and their uses. Their rules and modes of reasoning and practices, their logic applied (with a little more rubber) would justify the footpad and highwayman. Two cases will illustrate what I mean:

Some years since I chanced to visit the office of a leading dentist in a neighboring city. Immediately after me there came a young woman to have a tooth filled. The doctor commenced work at once, asking me not to leave. By a few strokes of an excavator, not even a "wipe out" with absorbent cotton, no antiseptic agent, no coffer dam; a lower molar was prepared for filling. Pouring some alloy in the palm of his hand from a white glass bottle, clear to the light, he amalgamated the alloy; squeezed the resultant mass between his thumb and finger, and "stuffed" the amalgam in the cavity, wiping the surplus away with his thumb, named two dollars as his fee, caressingly folded the bill paid and put it in his pocket. The proceeding did not occupy more than five to eight minutes. After the patient left I inquired, "Do you not use the dam in filling with amalgam?" "Oh, no," he answered. "I just hoe out the cavity and slap in the amalgam." I said: "But such a filling will only last a short time." "I know it, but she will come back and I will get another two dollars." I cannot write out the chuckle emphasizing the rejoinder.

Four days previous to the date of this writing, a young woman came to my office for some dental work. A brief examination revealed results of such work as just described. Eleven cavities containing plugs from what I judged to be "white alloy," and out of the eleven not one suffiiciently filled its cavity to allow successful patching. Three of the teeth had dead pulps and open fistule. I inquired how long since the fillings had been inserted. She replied "A little more than a year." Her teeth were fair sized, well formed and in good position.

Another case (an interruption while writing the last above). A young lady about twenty-two years of age, called to see if I could fill a front tooth with gold. Noting my surprise at her question, she explained that her dentist said her tooth would not hold gold. Looking at her teeth I saw two lower ones filled with amalgam. Both fillings were defective, one so much so that I easily passed a broach beside the plug to the bottom of a rather deep cavity. The other was only held in place by gravitation and wedging particles of food. I learned that the two fillings had been inserted about three months previously. They were "white alloys" amalgamated, but utterly worthless, as tooth preservatives. A strange part of her case was that the same dentist had "bridged" in a couple of superior bicuspids quite artistically and durably

But why multiply cases? I doubt if there is a dentist that will read this article but could multiply the three cases cited by almost any numeral, and then the half not be told. Dentists know that the large majority of amalgam fillings are faulty. I had almost said dismal failures as preservatives. Are they necessarily so? By no means. A careful scrutiny of any number of faulty fillings (amalgam) will reveal the cause of their worthlessness. In some portions of most of them there is solidity, close adaptation to dental walls and sufficient density. But like an anchor chain with a flawed link, they are a delusion, The fault does not lie in the alloys, bnt to the amalgamating and manipulating; the *lady*, *shiftless*, *dishonorable* and *dishonest* workmanship. Herein lies the abuse. There are honorable men in our profession whose daily and yearly work prove what I say.

Without such workers amalgam could not have driven back its enemies (pretended friends being the worst) and maintained the rank it now securely holds.

When Prof. A. O. Hunt, of the Dental Department of Iowa University, demonstrated in his clinic before the Chicago Dental Society, a few years since, that no more free mercury could be brought to the surface of his work; he gave the unanswerable argument in favor of the use of amalgams, and an object lesson revealing its abuse.

Everybody knows that free mercury is not a good preservative of tooth structure. Prof. Hunt taught how to divest the amalgamated alloys of their mercurial supersaturation and brushed aside the fine spun theories of "spheroidal tendency" of amalgam, crevicing around an amalgam filling, bulging of its surface, tendency to spherical form, ovoid tendency, etc., and demonstrated that if the superfluous mercury were gotten rid of and the mass packed so that equal density prevailed throughout and left to crystalize in that position, it would not "hump" or "globulate."

Could Dr. Hunt so condense amalgam in a tooth in the mouth without the aid of a coffer dam or some other method of dryness? If not, then no further argument is needed to point out one abuse of amalgam, namely, "submarine working." Another abuse is nearly or quite universal.—*Dental Review.*

